# The effect of high-intensity interval training on health-related outcomes in obese adolescents: a systematic review and meta-analysis

**DOI:** 10.3389/fphys.2025.1609818

**Published:** 2025-08-20

**Authors:** Gang Xu, Qiang Li, Qian Yang, Hongli Yu

**Affiliations:** ^1^School of Physical Education, Sichuan University of Science and Engineering, Zigong, China; ^2^Department of Kinesiology, University of Georgia, Athens, GA, United States; ^3^School of Physical Education, Shangrao Normal University, Shangrao, China; ^4^Department of Library, Sichuan University of Science and Engineering, Zigong, China

**Keywords:** high-intensity interval training, health-related outcomes, obese adolescents, systematic review, meta-analysis

## Abstract

**Objective:**

The present research was to evaluate the impacts of high-intensity interval training (HIIT) intervention on health-related outcomes in obese adolescents, adopting a systematic review and meta-analysis.

**Methodology:**

The study was performed by searching four databases (PubMed, Web of Science, Embase, and Cochrane Library) to determine randomized controlled trials (RCTs) exploring the impacts of HIIT to physiological parameters among obese adolescents. The sequential execution of a meta-analyses, subgroup analyses, and publication bias analyses was accomplished utilizing the software package RevMan version 5.4 and Stata 18.

**Results:**

There were 11 articles included. This research demonstrated a significant impact on body fat percentage (BFP) with d = −0.82, P < 0.05; VO_2_peak with d = 2.99, P < 0.05; high density lipoprotein (HDL) with *I* ^2^ = 22.8%, SMD = 0.47, 95% CI [0.06 to 0.88]; systolic blood pressure (SBP) with *I* ^2^ = 0.3%, SMD = −0.93, 95% CI [−1.25 to −0.62]. However, there was lacking of significance of body mass index (BMI) d = −0.21, P = 0. The subgroup analyses revealed that HIIT interventions involving with twice per week, 30–60 min per session, 1–30 min per session were the most effective in improving BMI, BFP and VO_2_peak.

**Conclusion:**

HIIT intervention, its impact on BMI is minimal, HIIT significantly reduces BFP, enhances VO_2_peak, increases HDL levels, and lowers SBP. HIIT effectively enhances body composition and cardiovascular health in overweight adolescents. Future studies should aim to refine HIIT protocols and explore its long-term benefits to establish comprehensive exercise recommendations for this population.

**Systematic Review Registration:**

identifier CRD42025630480.

## 1 Introduction

The increasing prevalence of adolescent obesity has emerged as a critical worldwide health issue. In 2022, more than 390 million individuals aged 5–19 were identified as overweight. The proportion of overweight and obese individuals in this demographic has skyrocketed, jumping from just 8% in 1990 to a staggering 20% in 2022 (Bouamra et al., 2022; WHO, 2025). Adolescence, a pivotal stage characterized by significant physical and psychological development (Thivel et al., 2016), is particularly sensitive to the adverse effects of obesity (de Fátima Aguiar Lopes et al., 2021; Su et al., 2024), including cardiovascular diseases, metabolic disorders, plus psychological issues like depression and low self-esteem (Buchan et al., 2010; Manojlovic et al., 2023; Martin-Smith et al., 2019; Inoue et al., 2020; Li and Chen, 2021; Khalafi et al., 2024).

Multiple studies have evaluated the effectiveness of interventions targeting obese adolescents. A systematic review revealed that dietary, physical activity, and behavioral interventions effectively reduced body mass index (BMI) and body fat percentage (BFP) in obese or overweight adolescents aged 11 to 19 (Al-Khudairy et al., 1996). Moreover, mobile health (mHealth) smartphone-based interventions have demonstrated positive impacts on weight management for both overweight adolescents and adults (Metzendorf et al., 2024). Another study highlighted that motivational interviewing combined with peer involvement yielded significant improvements in adolescent obesity management (Wu et al., 2016).

Based on these findings, high-intensity interval training (HIIT) has emerged as a complementary approach with substantial potential to enhance intervention outcomes. HIIT, which involves brief episodes of vigorous exercise interspersed with lower-intensity recovery phases, has attracted significant interest as an efficient and effective approach to enhancing health outcomes in diverse populations (Herget et al., 2016). In contrast to conventional moderate-intensity continuous training (MICT), HIIT has demonstrated comparable or even greater improvements in cardiorespiratory fitness, body composition, and metabolic health, all while demanding a reduced time commitment (Martin-Smith et al., 2020; Dias et al., 2016). This makes HIIT particularly appealing for adolescents, who may face barriers to prolonged exercise due to school schedules, social commitments, or lack of motivation (Domaradzki et al., 2020; Paulino da Silva Bento et al., 2021).

HIIT has been demonstrated to enhance multiple physiological parameters, such as endurance, cardiorespiratory fitness, and engagement in exercise among healthy adolescents. In addition to its established benefits on body composition and cardiovascular fitness, recent research has begun to explore the molecular and biochemical impacts of high-intensity training interventions. Several studies have demonstrated that HIIT or other high-intensity modalities, particularly when combined with nutritional supplementation, can improve inflammatory and metabolic markers. For example, astaxanthin-supplemented high-intensity training was shown to reduce adipokines and cardiovascular risk factors in men with obesity (Saeidi et al., 2023), while spirulina or Zataria multiflora supplementation alongside HIIT reduced tumor necrosis factor-alpha (TNF-α) and retinol binding protein-4 (RBP4), suggesting a systemic anti-inflammatory effect (Tayebi et al., 2018). Additionally, improvements in novel adipokines such as omentin-1 and lipocalin-2 have been reported following HIIT interventions (Atashak et al., 2022), and exercise training has been linked to the modulation of neuregulin 4, a key factor in obesity-related metabolic regulation (Tayebi et al., 2017). These mechanistic findings further highlight the broader potential of HIIT beyond traditional physiological outcomes, and underscore the need to incorporate such biomarkers in future research involving obese youth. Another comprehensive review has indicated that HIIT interventions lead to notable enhancements in VO_2_max, diastolic and systolic blood pressure, as well as maximum heart rate among healthy adolescents and children (Men et al., 2023). While numerous studies have explored the effects of low-to moderate-intensity exercises on BMI, blood pressure, and body fat in obese adolescents, the results regarding the efficacy of HIIT for promoting body composition in this population are mixed. A meta-analysis concluded that HIIT leads to favorable body composition outcomes, including reductions in body fat percentage and fat mass, as well as improvements in fat-free mass (Khodadadi et al., 2023). However, other meta-analyses have indicated that HIIT does not produce significant differences in reducing BFP or promoting overall body composition in overweight adolescents (Poon et al., 2024). The inconsistencies in these findings highlight the need for additional studies to determine HIIT’s precise impact on obese adolescents. Existing studies indicate: 1) mixed findings on HIIT’s impact across diverse physiological measures; 2) a lack of systematic reviews and meta-analyses focused on the Impact of HIIT on obese adolescents; and 3) a lack of comprehensive analyses of how HIIT influences specific physiological parameters in this population.

This study aims to perform a systematic review and meta-analysis evaluating HIIT’s impact on health-related outcomes in obese adolescents. By integrating findings from observational studies and randomized controlled trials (RCTs), it seeks to address a crucial question: To what extent does HIIT enhance physiological parameters. Additionally, the study will shed light on current gaps in the existing body of research and provide actionable insights to inform the creation of specialized interventions for this particular demographic.

## 2 Materials and methods

### 2.1 Protocol

Our methodology adheres to the guidelines outlined in the Preferred Reporting Items for Systematic Reviews and Meta-Analyses extension for Meta-Analyses (PRISMA-MA) within the healthcare field. Furthermore, it complies with the Cochrane Collaboration standards. These methodological frameworks have been comprehensively documented in foundational studies conducted by previous researchers (Higgins et al., 2011a; Page et al., 2021). Additionally, the protocol for this meta-analysis has been officially registered with PROSPERO, bearing the registration number (CRD42025630480), ensuring adherence to established standards for the registration and reporting of systematic reviews.

### 2.2 Search strategy and study selection

Two independent researchers meticulously scoured four major electronic databases (PubMed, Web of Science, Embase, and Cochrane Library) spanning records from their inception up to 20 December 2024. The investigation was guided by the PICOS framework (Population, Intervention, Comparator, Outcomes, and Study Design), which helped streamline the search strategy (Supplementary Material S1–S4). The selection of outcome variables in this meta-analysis was based on both the frequency with which they were reported across eligible studies and their established clinical relevance to obesity-related health risks in adolescents. Parameters such as BFP, BMI, VO_2_peak, HDL, and SBP are widely recognized and commonly used in obesity intervention research involving youth populations. These indicators provide insight into cardiometabolic health and physical fitness and are often prioritized in clinical and public health guidelines evaluating exercise effectiveness (Migueles et al., 2023; Dobbins et al., 2013; Lloyd et al., 2014). The study design was limited to RCTs. The search strategy incorporated keywords in conjunction with Boolean operators to refine the retrieval process (High Intensity Interval Training OR High-Intensity Interval Trainings OR Interval Training, High-Intensity OR Interval Trainings, High-Intensity OR Training, High-Intensity Interval OR Trainings, High-Intensity Interval OR High-Intensity Intermittent Exercise OR Exercise, High-Intensity Intermittent OR Exercises, High-Intensity Intermittent OR High-Intensity Intermittent Exercises OR Sprint Interval Training OR Sprint Interval Trainings OR HIIT) AND (Obesity OR obese OR fat OR corpulence OR adiposis OR overweight) AND (Adolescent or adolescents or adolescence or youth or youths or teens or teenager) AND (RCTs). The complete research approach is available in Supplementary Table S1. To ensure thorough coverage of the literature, a meticulous manual search was carried out to pinpoint pertinent systematic reviews, meta-analyses, key international conferences, and references from the studies included. Two independent investigators, whose identities were kept under wraps to maintain impartiality, conducted the initial screening of study results. This process was streamlined using Endnote software (version 21, Thompson ISI ResearchSoft), enabling the systematic identification and removal of duplicate records to enhance dataset reliability. Subsequently, two blinded investigators performed a full-text eligibility assessment, resolving any disagreements through deliberation or by seeking input from the third expert.

### 2.3 Inclusion and exclusion criteria

The criteria for study eligibility in the meta-analysis were determined by assessing key factors, including population characteristics, intervention type, comparator conditions, outcome measures, and study design. The inclusion criteria encompassed the following:

1. Adolescents aged 11–19 years classified as overweight or obese, without coexisting medical conditions.
2. Interventions involving HIIT, which includes various forms of exercise such as cycling and running, delivered in person.
3. Standard care will be the comparisons.
4. Availability of health outcome data.
5. RCTs, whether employing parallel or crossover frameworks, were included without imposing limitations based on criteria such as obesity classification, geographic region, ethnic background, language, or publication date. Existing scholarly definitions were leveraged to classify interventions as mHealth in-person, and eHealth approaches.

Exclusion criteria included: 1) studies involving non-human subjects; 2) interventions other than HIIT; 3) unavailable data; and 4) protocols or studies not employing RCTs.

### 2.4 Data collection and management

The extraction of data from the selected RCTs was carried out independently by two researchers, who remained blinded to minimize bias. A standardized data collection form, developed in accordance with the guidelines established by the Cochrane Consumers and Communication Review Group, was employed to ensure consistency and accuracy in data recording (Chandler et al., 2019). The dataset compiled consisted of several key elements: (1) Core article information, encompassing the lead author’s name, the study’s geographical setting, and publication date; (2) Salient demographic data, including the participants’ age range, the specific population under examination, and the overall number of subjects involved; (3) A breakdown of participant assignment to either the intervention or control arms, a detailed description of the procedures implemented in the intervention group, and a clear outline of the stipulations imposed on the control group; (4) Variables related to physical activity, encompassing exercise modality, training cycle, session frequency, and duration. Data extraction for the general characteristics of the eight selected meta-analyses was conducted by two independent reviewers using a standardized data collection form. Any inconsistencies identified during this process were addressed through dialogu or, if necessary, by consulting a third expert.

### 2.5 Quality appraisal

The quality of the RCTs we selected was evaluated using the Cochrane Collaboration’s Risk of Bias (ROB) tool, sticking closely to the instructions laid out in study (Shuster, 2011). The assessment of each RCT was conducted independently by two researchers. Following the guidelines specified in the Cochrane Handbook 5.1.0, the evaluation primarily targeted research bias, incorporating seven distinct criteria: 1) How the random sequence was generated (selection bias); 2) Allocation concealment (also selection bias); 3) Whether the participants and staff were blinded (performance bias); 4) Blinding of the outcome assessors (detection bias); 5) Incomplete outcome data (attrition bias); 6) Selective reporting (reporting bias); and 7) any other sneaky biases. Based on these criteria, the studies were then slotted into one of three categories: “low risk,” “unclear,” or “high risk.” Initially, each study got a once-over from two researchers. If there was a disagreement, a third party stepped in to break the tie. We used RevMan (Version 5.4, The Cochrane Collaboration, 2020) to conduct the ROB assessment.

### 2.6 Statistical analysis

Stata 18 statistical software was employed in this study to integrate effect sizes and evaluate potential biases. Inconsistencies were noted in how depression indicators were measured in the original studies analyzed, aiming to improve the precision of effect size estimation, all data were standardized using standardized mean difference (SMD), weighted mean difference (WMD), and 95% confidence intervals (CI). The SMD was derived by computing the difference between pre- and post-intervention mean values and dividing it by the final pooled standard deviation (SD). This method addresses the problem of inconsistent measurement units across various scales. Based on the *I* ^2^ Range, we take 0%–25% as low heterogeneity, indicating that there is little difference between the study results, and heterogeneity may not significantly affect the interpretation of the findings. 25%–50%: Moderate heterogeneity, suggesting that there is some variation between studies, and the potential sources of heterogeneity should be further explored. 50%–75%: High heterogeneity, indicating considerable differences between studies, and more attention is required to understand potential causes of the variation, such as study design, sample characteristics, *etc.*, 75%–100%: Very high heterogeneity, meaning there are significant differences between studies. This suggests the presence of potential biases or confounding variables, and careful interpretation of the results is needed. To assess potential publication bias, a funnel plot was examined, and Egger’s regression analysis was performed.

## 3 Results

### 3.1 Search results and study characteristics

**FIGURE 1:**
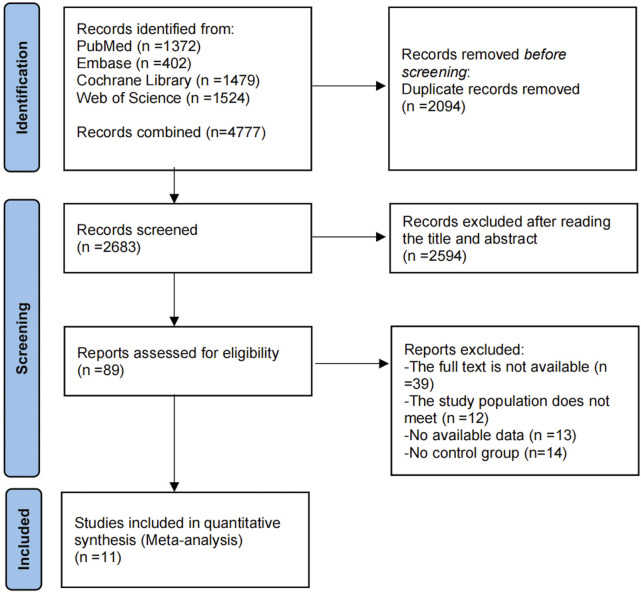
PRISMA diagram depicting the sequential steps of the selection process.

The PRISMA-MA flowchart illustrating the literature selection process is presented in Figure 1. An initial search yielded 4,777 publications. After removing 2,094 duplicate entries, 2,683 articles were excluded based on the screening of titles and abstracts. A comprehensive full-text assessment was then performed on 89 studies that appeared to meet the eligibility criteria. Among these, 78 were excluded for various reasons: 13 due to insufficient data, 39 because the full text was unavailable, 12 for failing to meet the inclusion criteria, and were omitted due to the lack of a control group. Ultimately, the analysis encompassed 11 studies, comprising 611 adolescents classified as overweight or obese. The study involved 611 participants in total, with 328 comprising the experimental group and 283 in the control. The ages of the participants varied between 11 and 17 years. The research cast a wide net geographically, encompassing Poland, Denmark, and Spain in Europe; China in Asia; South Africa; and the U.S. and Brazil in the Americas. All of included studies are RCTs, published in English between 2014 and 2024. Table 1 provides detailed participant and intervention characteristics.

**TABLE 1:** Summary of features of included intervention.

First author year	Participants characteristics					Intervention characteristics				Control group	Outcome
	Region	Study design	Target sample	Age	Sample size (N)	Intervention description	Intervention length	Duration	Frequency		
Boer et al. (2014)	South Africa	RCT	Secondary school students	17.4 ± 2.4	I:17 C: 14	Sprint interval training-cycling	7 weeks	10 min	2 sessions/week	Routine Prenatal care	BMI, BFP, SBP, HDL
Cao et al. (2022)	China	RCT	Grade school students	11.2 ± 0.7	I: 20 C: 20	Sprint interval training-20-m shuttle run test	4 weeks	60 min	1 session/week	Routine Prenatal care	BMI, BFP, VO_2_peak
Domaradzki et al. (2022)	Poland	RCT	Secondary school students	16.21 ± 0.31	I: 31 C: 21	High-Intensity Interval Training (HIIT)	10 weeks	14 min	1 session/week	Routine Prenatal care	BMI
Eggertsen et al. (2024)	Denmark	RCT	Obesity clinics	12.3 ± 1.7	I: 90 C: 83	High-Intensity Interval Training (HIIT)	12 weeks	45 min	3 sessions/week	Routine Prenatal care	BMI
González-Gálvez et al. (2024a)	Spain	RCT	Secondary school students	12.51 ± 0.75	I: 20 C: 20	High-Intensity Interval Training (HIIT)	8 weeks	12 min	2 sessions/week	Routine Prenatal care	BMI, BFP, VO_2_peak, SBP, HDL
González-Gálvez et al. (2024a)	Spain	RCT	High school students	12.51 ± 0.75	I: 15 C: 15	High-Intensity Interval Training (HIIT)	8 weeks	5 min	2 sessions/week	Routine Prenatal care	BMI, BFP, VO_2_peak, SBP, HDL
Meng et al. (2022)	China	RCT	Experimental School in China	11.2 ± 0.7	I: 12; C: 13	High-Intensity Interval Training (HIIT)	12 weeks	5 min	3 sessions/week	Routine Prenatal care	BMI, BFP, VO_2_peak, SBP, HDL
Su et al. (2024)	China	RCT	A weight loss camp	14 ± 1	I: 22 C: 22	High-Intensity Interval Training (HIIT)	8 weeks	45 min	3 sessions/week	Routine Prenatal care	BMI, VO_2_peak
Tas et al. (2023)	United States of America	RCT	The Arkansas Children’s Hospital	15.2 ± 1.5	I: 31 C: 6	High-Intensity Interval Training (HIIT) intervention	4 weeks	45 min	1 session/week	Routine Prenatal care	BMI, VO_2_peak, HDL
Tian et al. (2021)	China	RCT	Middle schools students	12.3 ± 0.5	I: 44 C: 43	high-intensity interval exercise (HiInEx)	24 weeks	10 min	3 sessions/week	Routine Prenatal care	BMI, BFP
Faria et al. (2020)	Brazil	RCT	Public schools	15.5 ± 0.9	I: 26 C: 26	high-intensity interval exercise-Running	12 weeks	20 min	3 sessions/week	Routine Prenatal care	BMI, VO_2_peak, SBP

Abbreviations: I, intervention; C, control; BMI, body mass index; VO_2_peak, peak oxygen consumption; BFP, body fat percentage; SBP, systolic blood pressure; HDL, high-density lipoprotein.

### 3.2 Quality evaluation

Flaws in trial design, execution, analysis, and reporting can impede the precise determination of causal links. Such limitations can result in either an overestimation or underestimation of the actual effect of the intervention, thereby introducing bias into the findings (Wood et al., 2008). However, it is typically challenging to pinpoint the exact impact of biases on the results of a specific trial (Higgins et al., 2011b). The main aim of employing the Cochrane Risk of Bias Tool is to assess the methodological soundness and uncover potential biases in medical studies, particularly in RCTs. Developed by the Cochrane Collaboration, this tool assists researchers, clinicians, and policymakers in recognizing biases that could compromise the validity of study outcomes. The assessment focuses on six specific types of bias: selection, detection, performance, reporting, attrition, and other potential sources of bias (Chandler et al., 2019).

**FIGURE 2:**
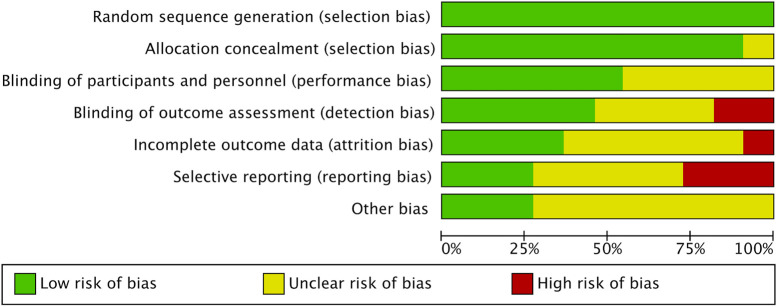
Risk of Bias graph.

**FIGURE 3:**
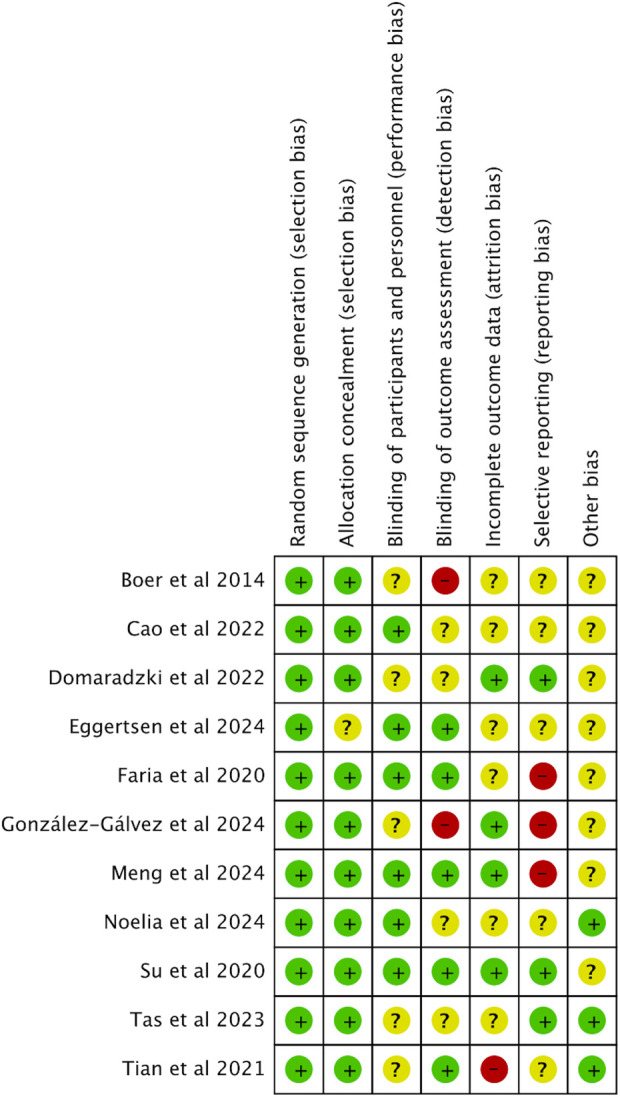
Risk of bias summary.

This analysis exclusively included randomized controlled trials (RCTs). When it comes to the random sequence generation, over half the studies, to be precise, 54.4%, showed minimal bias risk overall, yet allocation concealment issues were noted in 37.7% of the studies (Cao et al., 2022; Domaradzki et al., 2022; Eggertsen et al., 2024; González-Gálvez, López-Gil, et al., 2024a; González-Gálvez, Soler-Marín, et al., 2024b; Tas et al., 2023; Faria et al., 2020). Performance bias, specifically concerning participant and personnel blinding, was significantly high in 8.8% of the studies (Boer et al., 2014; González-Gálvez, López-Gil, et al., 2024a; Tian et al., 2021). Additionally, 9% of the studies exhibited a significant level of attrition bias, while reporting bias was assessed as high in 2.3% of cases. Other biases, mainly associated with small sample sizes, were considered problematic in 20.7% of the studies. Figures 2, 3 provide detailed ROB assessments at both the individual and overall study levels.

### 3.3 Meta-analysis and tests for bias

#### 3.3.1 Effect of HIIT on BMI of obese adolescents

**FIGURE 4:**
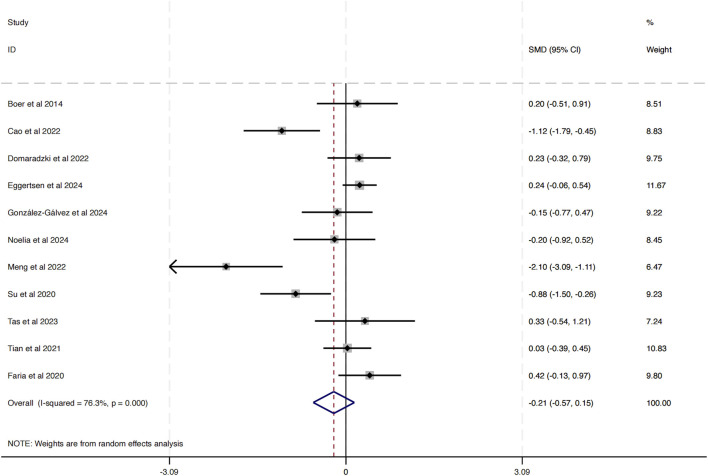
Forest plot illustrating the impact of HIIT on BMI in obese adolescents.

A total of 11 studies were incorporated into this analysis. As illustrated in Figure 4, the results revealed substantial heterogeneity among the included studies (*I* ^2^ = 76.3%, P = 0.00). The analysis of the overall effect size yielded statistically significant findings (SMD = −0.21, 95% CI [−0.75 to −0.15]), indicating that HIIT does not exert a notable influence on BMI in obese adolescents.

**FIGURE 5:**
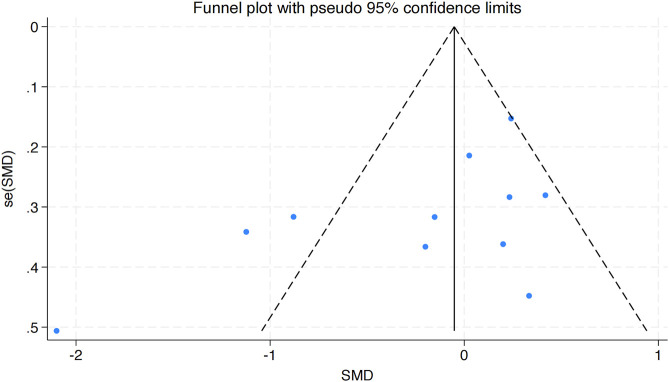
Bias funnel plot of HIIT on BMI in obese adolescents.

The funnel plot in Figure 5 analysis showed that the scatter points were evenly distributed within the funnel, indicating no significant publication bias. To further assess this, an Egger’s test was conducted for BMI, yielding Pr > |z| = 0.076, which is greater than 0.05, verifying the lack of significant publication bias.

##### 3.3.1.1 Subgroup analyses

Given the substantial heterogeneity observed in the meta-analysis of HIIT’s effects on obese adolescents, a subgroup analysis in Supplementary Figure S1 was performed for this parameter. Considering the potential influence of exercise frequency, Researches was classified into three separate categories.

• **Group 1**: Exercising once per week (comprising 3 studies)
• **Group 2**: Exercising twice per week (comprising 3 studies)
• **Group 3**: Exercising three times per week (comprising 5 studies)

The subgroup analysis revealed that the group exercising twice per week exhibited the lowest heterogeneity (*I* ^2^ = 0.0, P = 0.701). However, the subgroup meta-analyses of exercise duration per session and weekly exercise frequency both exhibited high heterogeneity. These findings suggest that the variability in HIIT’s impact on BMI among obese adolescents may be attributed to differences in weekly exercise frequency.

#### 3.3.2 Effect of HIIT on BFP of obese adolescents

**FIGURE 6:**
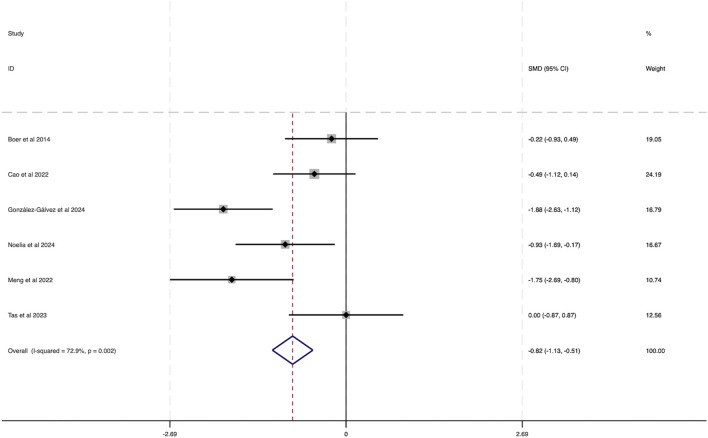
Forest plot illustrating the impact of HIIT on BFP in obese adolescents.

Six studies were incorporated into this analysis. As shown in Figure 6, considerable heterogeneity was observed across the included research (*I* ^2^ = 72.9%, P = 0.002). The evaluation of the combined effect size yielded statistically meaningful outcome (SMD = −0.82, 95% CI [−1.13 to −0.51]), indicating the substantial influence of HIIT on lowering BPF among obese adolescents, thereby contributing to health benefits.

**FIGURE 7:**
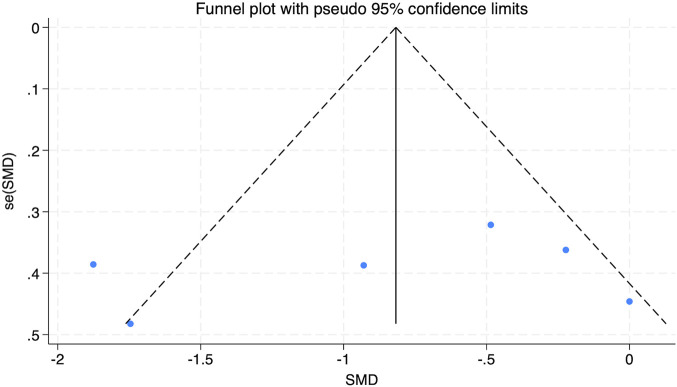
Bias funnel plot of HIIT on BFP in obese adolescents.

The funnel plot in Figure 7 analysis showed that the scatter points were evenly distributed within the funnel, indicating no significant publication bias. Additionally, Egger’s test for BFP yielded a Pr > |z| value of 0.542, greater than 0.05, further indicating a lack of considerable publication bias.

##### 3.3.2.1 Subgroup analyses

Given the substantial heterogeneity observed in the meta-analysis of HIIT’s effect on BFP in obese adolescents, a subgroup in Supplementary Figure S2 analysis was conducted to explore potential sources of this variability. Considering the possible impact of exercise duration, studies were categorized into two groups based on session length.

• **Group 1**: Sessions lasting 1–30 min (comprising four studies)
• **Group 2**: Sessions lasting 30–60 min (comprising two studies)

The subgroup analysis revealed that the heterogeneity was lowest in the 30–60 min per session group (*I* ^2^ = 0.0%, P = 0.377). However, the subgroup meta-analyses of weekly exercise frequency and total weeks of exercise both exhibited high heterogeneity. These findings suggest that the variability in HIIT’s effect on BFP among obese adolescents could be linked to the length of individual exercise sessions.

#### 3.3.3 Effect of HIIT on VO_2_peak of obese adolescents

**FIGURE 8:**
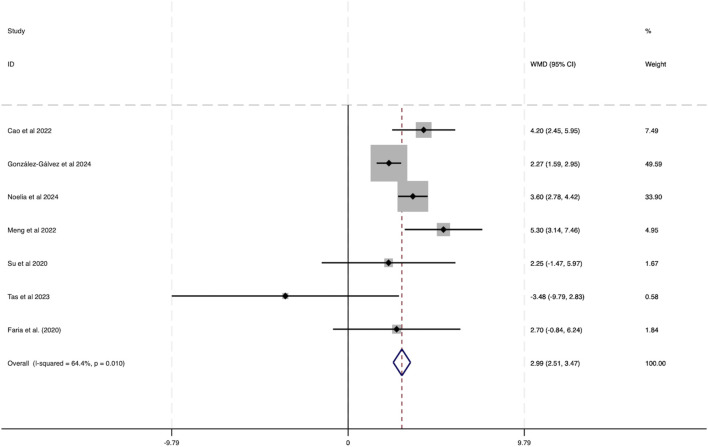
Forest plot illustrating the impact of HIIT on VO_2_peak in obese adolescents.

This analysis integrated data from seven studies, as depicted in Figure 8. The results demonstrated considerable heterogeneity among the included studies (*I* ^2^ = 64.4%, P = 0.010). A statistically significant combined effect size was observed (WMD = 2.99, 95% CI [2.51 to 3.47]), indicating that HIIT exerts a notable positive effect on VO_2_peak in obese adolescents, thereby contributing to improved health outcomes.

**FIGURE 9:**
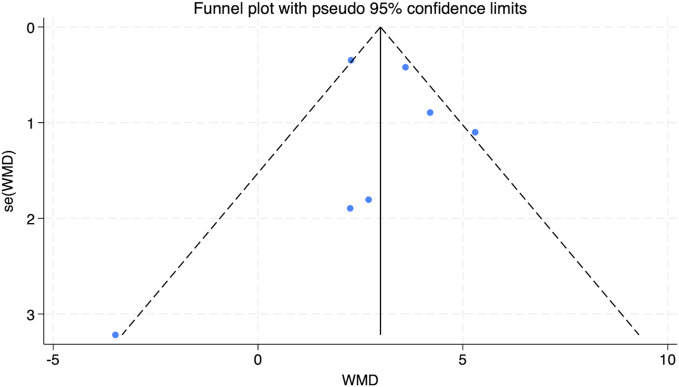
Bias funnel plot of HIIT on VO_2_peak in obese adolescents.

The funnel plot analysis in Figure 9 revealed a symmetrical distribution of data points, suggesting no evident publication bias. This finding was further corroborated by Egger’s test for VO_2_peak (Pr > |z| = 0.928), which exceeded the 0.05 threshold, firming the lack of meaningful publication bias.

##### 3.3.3.1 Subgroup analyses

Given the considerable heterogeneity observed in the meta-analysis of HIIT’s effect on Vo_2_peak among obese adolescents, a subgroup analysis was performed to investigate possible factors contributing to this variability. Duration of each exercise session was considered as a moderating factor. Studies were categorized into two groups based on session length.

• **Group 1:** Sessions lasting 1–30 min (comprising four studies).
• **Group 2:** Sessions lasting 30–60 min (comprising two studies).

The subgroup in Supplementary Figure S3 analysis revealed that the 1–30 min session group exhibited the lowest heterogeneity (*I* ^2^ = 0.0%, P = 0.399). However, the subgroup meta-analyses of weekly exercise frequency and total weeks of exercise both exhibited high heterogeneity. These findings suggest that the variability in HIIT’s effect on Vo_2_peak among obese adolescents may stem from variations in exercise session length.

These results underscore the importance of tailoring HIIT protocols, particularly concerning session duration, to effectively enhance cardiorespiratory fitness in obese adolescent populations.

#### 3.3.4 Effect of HIIT on high density lipoprotein (HDL) of obese adolescents

**FIGURE 10:**
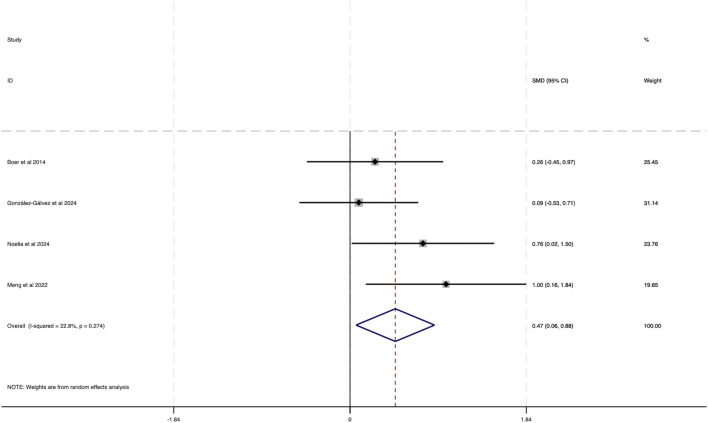
Forest plot illustrating the impact of HIIT on HDL in obese adolescents.

This analysis integrated data from four studies, as shown in Figure 10. The results indicated a low degree of heterogeneity among the included studies (*I* ^2^ = 22.8%, P = 0.274). A statistically significant combined effect size was observed (SMD = 0.47, 95% CI [0.06 to 0.88]), suggesting that HIIT exerts a beneficial effect by increasing HDL levels in obese adolescents, which contributes to improved health.

**FIGURE 11:**
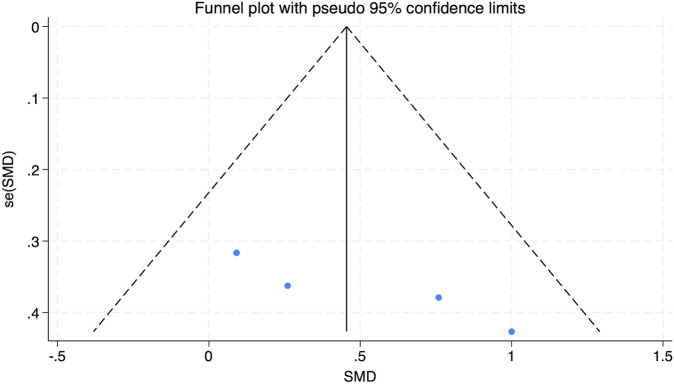
Bias funnel plot of HIIT on HDL in obese adolescents.

The funnel plot analysis in Figure 11 displayed an even distribution of data points within the funnel, implying no substantial publication bias. Furthermore, Egger’s test for HDL yielded a Pr > |z| value of 0.064, surpassing the 0.05 threshold, thereby supporting the absence of significant publication bias.

### 3.3.5 Effect of HIIT on systolic blood pressure (SBP) of obese adolescents

**FIGURE 12:**
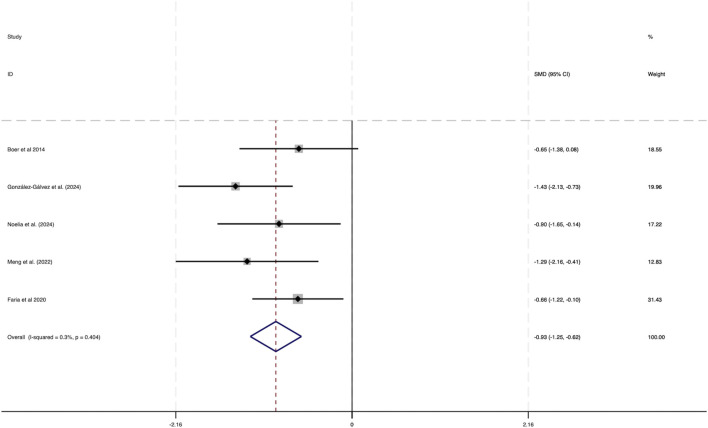
Forest plot illustrating the impact of HIIT on SBP in obese adolescents.

Data from five studies were analyzed in this section, as depicted in Figure 12. The findings revealed minimal heterogeneity across the included studies (*I* ^2^ = 0.3%, P = 0.404). A statistically significant combined effect size was obtained (SMD = −0.93, 95% CI [−1.25 to −0.62]), indicating that HIIT plays a crucial role in lowering SBP among obese adolescents, thereby enhancing overall health.

**FIGURE 13:**
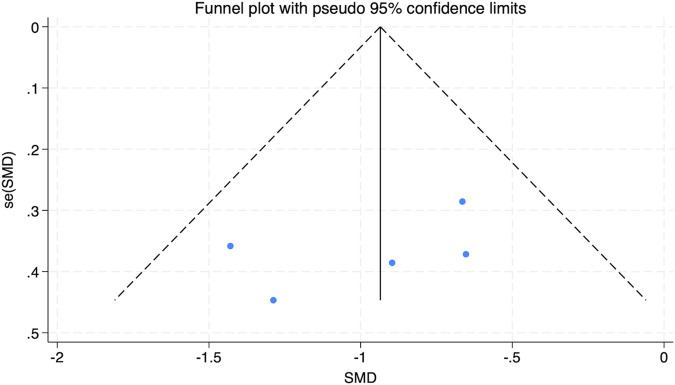
Bias funnel plot of HIIT on SBP in obese adolescents.

The funnel plot analysis presented in Figure 13 showed a symmetrical distribution of data points, suggesting no meaningful publication bias. Additionally, Egger’s test for SBP resulted in a Pr > |z| value of 0.340, which exceeded 0.05, further substantiating the lack of notable publication bias.

### 3.4 Sensitivity analyses

To further assess the robustness of the meta-analysis findings, sensitivity analyses were conducted for BMI, VO_2_peak, and BFP by sequentially omitting one study at a time (leave-one-out method). The results, illustrated in Supplementary Figure S4, demonstrated that no single study significantly altered the overall effect size estimates for any of the three outcomes. This suggests that the findings are stable and not driven by any individual study, including those with smaller sample sizes or potentially higher risk of bias. These results enhance confidence in the overall conclusions, despite the high heterogeneity observed in the pooled estimates.

## 4 Discussion

This systematic review and meta-analysis examined the effects of HIIT on key health-related outcomes in obese adolescents. A total of 11 RCTs involving 611 participants aged 11–17 years were included. The findings indicate that while HIIT does not significantly impact BMI, it has substantial effects on reducing BFP, improving VO_2_peak, enhancing HDL levels, and lowering SBP. These results underscore the potential of HIIT as an effective intervention for improving body composition and cardiovascular health in obese adolescents, despite its limited effect on BMI.

The studies included in our analysis primarily focused on adolescent populations, with sample sizes ranging from small experimental school-based cohorts (e.g., Cao et al., 2022; Meng et al., 2022) to larger obesity clinic populations (e.g., Eggertsen et al., 2025). The target age groups varied across studies, with most participants falling within the range of 11–17 years. For the small sample size, it is the potential concern regarding the impact of HIIT on adolescent development, adolescence is a pivotal growth phase marked by notable physiological and hormonal shifts that can impact the body’s reaction to HIIT (Rogol et al., 2002). Some of included studies showed some concerns of other bias in small sample sizes, which could amplify random errors and limit statistical power. Notably, studies included participants from specialized obesity clinics and schools, respectively, an important consideration is the variability in study settings, this distinction may have influenced the effectiveness and implementation of HIIT interventions.

The intervention environment is one of key factor which may impact on the effectiveness of intervention. Clinical settings typically have trained healthcare professionals, such as exercise physiologists, sports medicine specialists, or physiotherapists, who can tailor HIIT programs to individual needs, closely monitor participants, and provide immediate intervention if adverse effects arise (Song and Lan, 2024). This level of expertise may enhance adherence, safety, and the reliability of intervention outcomes. In contrast, school-based HIIT programs often rely on physical education teachers or general instructors, who may not have specialized training in exercise prescription for obese adolescents. As a result, variations in instruction quality, intensity regulation, and injury prevention strategies could lead to inconsistent outcomes across studies (Martin-Smith et al., 2020).

Additionally, geographic diversity was evident, with studies conducted in China, South Africa, the United States, Spain, Poland, Brazil, and Denmark, reflecting the global interest in HIIT as a potential strategy for addressing obesity-related health concerns in youth. A key observation is that studies from developed countries, particularly in Europe and North America, tended to implement standardized HIIT protocols with controlled intensity, structured progression, and professional supervision. These interventions often adhered to established exercise guidelines and incorporated advanced monitoring techniques, such as heart rate tracking and metabolic assessments, ensuring precise execution and evaluation of HIIT programs (Eddolls et al., 2018). In contrast, studies from developing regions, such as parts of Asia and Africa, demonstrated greater variation in intervention design, with some employing locally adapted HIIT programs that differed in duration, intensity, and supervision quality. These discrepancies may stem from differences in resource availability, access to professional training, and cultural attitudes toward structured exercise programs (Costigan et al., 2021).

Furthermore, socioeconomic factors and healthcare infrastructure may influence the effectiveness and sustainability of HIIT interventions. Adolescents in developed regions may have better access to fitness facilities, trained exercise professionals, and consistent follow-up assessments, enhancing adherence and long-term outcomes. Conversely, in lower-income settings, logistical barriers such as inadequate sports infrastructure, lack of trained personnel, and lower participant retention rates could limit the effectiveness of HIIT programs (Stavrinou et al., 2018).

The reviewed studies implemented various HIIT protocols, differing in training modality, intervention length, session duration, and frequency. The intervention frequency varied between 4 and 24 weeks. Short-term interventions (4–12 weeks) demonstrated rapid physiological improvements, including enhanced cardiorespiratory fitness, reduced adiposity, and improved metabolic markers (Eddolls et al., 2018). These benefits may be attributed to the immediate physiological responses to high-intensity exercise, such as increased mitochondrial biogenesis, improved insulin sensitivity, and enhanced fat oxidation (Weston et al., 2014). However, while short-term HIIT has been shown to elicit significant improvements, there are concerns regarding its long-term sustainability. Studies indicate that shorter training durations may not provide sufficient time for adolescents to establish habitual physical activity behaviors, potentially limiting the maintenance of these benefits post-intervention (Bond et al., 2017).

Conversely, longer intervention duration offer the advantage of progressive physiological adaptations and sustained behavioral changes. Prolonged HIIT programs allow for more substantial improvements in body composition, cardiovascular function, and metabolic health. Studies have shown that extended HIIT interventions contribute to greater reductions in visceral fat, improved lipid profiles, and enhanced muscular endurance (Gillen and Gibala, 2018). Furthermore, longer training periods may facilitate better adherence to exercise routines, fostering long-term engagement in physical activity (Stavrinou et al., 2018). However, the challenge with extended interventions lies in participant retention, as motivation may decline over time due to increased physical and psychological demands (Lubans et al., 2022).

Most of included studies utilized short term sessions. Shorter sessions were characteristic of sprint-based HIIT interventions (e.g., Cao et al., 2022; Boer et al., 2014; Meng et al., 2022), whereas moderate to longer sessions were employed in more traditional HIIT studies (e.g., Su et al., 2024; Tas et al., 2023). Short-duration HIIT sessions have gained popularity due to their efficiency and feasibility, particularly for adolescents with low exercise tolerance or limited time availability. Research indicates that short HIIT sessions can enhance cardiovascular health, insulin sensitivity, and fat metabolism (Sandbakk et al., 2013; Tjønna et al., 2009). The physiological benefits are primarily attributed to the high-intensity nature of the training, which induces rapid mitochondrial adaptations and enhances glucose uptake (MacInnis and Gibala, 2017). However, shorter sessions may limit total energy expenditure, which could reduce their effectiveness in promoting significant fat loss and long-term metabolic improvements (Buchan et al., 2013). Additionally, shorter training durations may not provide sufficient stimulus for substantial muscular adaptations compared to longer sessions.

Long term session durations may offer additional benefits by increasing total energy expenditure and promoting greater improvements in body composition and cardiovascular health. Studies indicate that longer training duration enhance fat oxidation, improve lipid profiles, and lead to greater reductions in body fat percentage (Weston et al., 2014; Viana et al., 2019). Furthermore, prolonged exercise sessions provide more opportunities for skill development, aerobic conditioning, and muscular endurance improvements. However, the increased physical demand of longer HIIT sessions may lead to higher dropout rates, particularly among obese adolescents who may experience greater fatigue and discomfort (Dias et al., 2018). Moreover, excessive session duration without proper recovery could increase the risk of over training and injury, potentially discouraging long-term participation in physical activity (Lubans et al., 2022).

Notably, a research studied by Tian et al. (2021) revealed that a 24-week, 3-sessions-per-week protocol resulted in notable enhancements in BMI and BFP, emphasizing the potential of long-term HIIT interventions in managing adolescent obesity. A minimal training frequency of once per week provides limited benefits, as high-intensity exercise is needed for long-term adaptations. A single HIIT session can improve insulin sensitivity and mitochondrial function (Little et al., 2010), but sustained benefits like fat reduction and aerobic capacity require more frequent training (Weston et al., 2014). Lower frequency hinders neuromuscular and cardiovascular adaptations, reducing fitness improvements in obese adolescents (Eddolls et al., 2017). Training twice weekly balances effectiveness and adherence, enhancing VO_2_ max, fat loss, and lipid profiles while minimizing overtraining risk (Logan et al., 2014). Increasing frequency to three times per week amplifies metabolic and cardiovascular benefits but raises fatigue and dropout risks (Ouerghi et al., 2017). Monitoring training load and ensuring recovery are crucial for effective HIIT implementation.

The most commonly assessed outcomes included BMI, BFP, VO_2_peak, SBP, and HDL levels in this study. The observed improvements in BMI and BFP across multiple studies highlight HIIT’s effectiveness in reducing adiposity among adolescents (e.g., Eggertsen et al., 2024; González-Gálvez et al., 2024b; Su et al., 2024). Previous studies have reported reductions in pro-inflammatory markers (e.g., TNF-α, RBP4), improvements in adipokines (e.g., lipocalin-2, omentin-1), and cardioprotective mechanisms involving neuregulin 4 and miR-206-mediated HSP60 expression following high-intensity training (Delfan et al., 2022; Supriya et al., 2023). Moreover, researches such as those by Meng et al. (2022) and Cao et al. (2022) demonstrated notable enhancements in VO_2_peak, indicating improvements in aerobic fitness. A notable reason in the included studies is the restricted number of physiological parameters assessed, with most studies reporting only two to three indicators. This reason may stem from the need to expand databases and the increased workload associated with incorporating a broader range of physiological measures. Comprehensive assessments, including metabolic markers, muscle composition, and cardiovascular parameters, would offer a fuller insight into the impacts of HIIT on obese adolescents (Song and Lan, 2024). Future studies should aim to incorporate a broader range of physiological indicators to enhance the robustness of findings.

The ROB evaluation showed that the majority of studies had minimal or intermediate bias risks. Few studies caused highest risks due to methodological limitations such as inadequate blinding, incomplete outcome data, and selective reporting, introduce biases that affect the reliability of conclusions. Additionally, inconsistencies in reporting all predefined primary outcomes reduce the clarity of HIIT’s impact. Future research should aim for standardized reporting, balanced sample sizes, and improved methodological rigor to better understand the optimal weekly frequency of HIIT for obese adolescents.

The findings from this meta-analysis indicate that HIIT alone has a limited impact on BMI of obese adolescents, despite conducting a subgroup meta-analysis, the high heterogeneity was associated with the frequency of exercise across different regions, a result consistent with previous studies. BMI may not be an ideal indicator for assessing the effectiveness of exercise interventions, as it fails to differentiate between adipose tissue and muscle mass (Tolfrey et al., 2014; García-Hermoso et al., 2018). However, our analysis shows that HIIT significantly improves BFP and VO_2_peak, making these parameters more reliable indicators of intervention effectiveness. Moreover, both of the subgroup meta-analysis indicated that the high heterogeneity was also related to the duration of each exercise session, both of which had significant effects. In addition to subgroup analyses, sensitivity tests were performed to evaluate the influence of individual studies on the pooled estimates. The leave-one-out approach confirmed that no single study disproportionately influenced the results for BMI, VO_2_peak, or BFP. This reinforces the robustness of the observed effects and suggests that the high heterogeneity was not primarily driven by outlier studies but may instead reflect broader variation in intervention design, sample characteristics, or delivery settings. Nonetheless, future trials with larger sample sizes and standardized reporting of protocol fidelity and bias risk are warranted to further validate these findings and reduce interpretive uncertainty. HIIT is known to promote fat loss while simultaneously increasing lean muscle mass (Callahan et al., 2021). Since BMI is a crude measure that does not differentiate between these components, reductions in fat mass may be offset by muscle gains, leading to minimal changes in BMI despite meaningful body composition improvements. In contrast, reductions in BFP provide a clearer reflection of the effectiveness of HIIT in reducing excess adiposity.

VO_2_peak, a marker of cardiovascular fitness, was also significantly improved following HIIT interventions. This aligns with previous studies demonstrating that HIIT enhances cardiorespiratory fitness through repeated exposure to high-intensity bouts, improving oxygen uptake efficiency (Buchan et al., 2013). Increased VO_2_peak is associated with better metabolic health, insulin sensitivity, and overall cardiovascular function, making it a critical outcome in assessing the benefits of HIIT in obese adolescents.

HIIT significantly enhances HDL cholesterol and SBP, demonstrating its vital impact on cardiovascular health in obese adolescents. The interaction between cardiovascular fitness, lipid profile, and blood pressure regulation is well-established, as VO_2_peak improvements are often accompanied by favorable changes in lipid metabolism and vascular function (Ramos et al., 2015). Enhanced VO_2_peak reflects increased cardiovascular efficiency, which may contribute to reductions in SBP through improved endothelial function and arterial compliance (da Silva et al., 2020).

Elevated HDL cholesterol levels correlate with a lower risk of cardiovascular disease, and HIIT has been shown to promote beneficial changes in lipid metabolism by increasing lipoprotein lipase activity and enhancing lipid transport (Scott et al., 2019). However, the effects of HIIT on LDL cholesterol were not consistently reported across the included studies, limiting our ability to assess its impact on this marker. Future research should aim to clarify HIIT’s role in modulating LDL and other lipid components to provide a more comprehensive understanding of its cardiovascular benefits. Additionally, reductions in SBP following HIIT interventions align with previous findings suggesting that high-intensity exercise improves autonomic regulation and arterial elasticity, contributing to better blood pressure control (Batacan et al., 2017). These results reinforce the potential of HIIT as an effective intervention for minimizing heart disease risks in obese adolescents.

### 4.1 Qualitative synthesis of HIIT protocol variability

Beyond the subgroup analyses, a qualitative comparison of the included studies revealed substantial variation in the structure and delivery of HIIT protocols, which may have contributed to the observed heterogeneity in outcomes. Interventions differed in session duration (ranging from 5 to 60 min), frequency (from once to three times per week), total intervention length (4–24 weeks), and exercise modality (e.g., sprint cycling, shuttle runs, bodyweight circuits, or running-based HIIT). Moreover, the delivery settings varied, with some studies conducted in structured environments such as clinics or weight-loss camps, while others were embedded within school physical education programs. These contextual and methodological differences likely influenced both the feasibility of implementation and the physiological adaptations observed.

To illustrate these differences more clearly, Table 2 presents a qualitative synthesis of the key features of each HIIT intervention, including modality, frequency, session duration, total length, and delivery setting. This comparative overview complements the quantitative subgroup analyses and provides practical insights into how specific protocol characteristics may relate to outcome variability. Future studies should consider aligning HIIT program design with participant context, setting-specific feasibility, and intended outcomes to ensure better standardization and generalizability.

**TABLE 2:** Qualitative comparison.

Study (first author, year)	Country	Setting	Modality	Session duration	Frequency	Total length	Notes
Boer et al. (2014)	South Africa	School	Sprint interval cycling	10 min	2×/week	7 weeks	Moderate duration
Cao et al. (2022)	China	School	Shuttle run	60 min	1×/week	4 weeks	Long session, low freq
Domaradzki et al. (2022)	Poland	School	HIIT	14 min	1×/week	10 weeks	Short session, once/week
Eggertsen et al. (2024)	Denmark	Clinic	HIIT	45 min	3×/week	12 weeks	Structured, clinical
González-Gálvez et al. (2024a)	Spain	School	HIIT	12 min	2×/week	8 weeks	PE integrated
González-Gálvez et al. (2024b)	Spain	School	HIIT	5 min	2×/week	8 weeks	Very short, high freq
Meng et al. (2022)	China	School	HIIT	5 min	3×/week	12 weeks	Very short, frequent
Su et al. (2024)	China	Weight-loss camp	HIIT	45 min	3×/week	8 weeks	Intensive setting
Tas et al. (2023)	United States of America	Hospital	HIIT	45 min	1×/week	4 weeks	Clinical setting
Tian et al. (2021)	China	School	HIInEx	10 min	3×/week	24 weeks	Extended duration
Faria et al. (2020)	Brazil	School	HIIT-running	20 min	3×/week	12 weeks	Moderate dose

### 4.2 Strengths and limitations

This systematic review and meta-analysis offer critical findings on HIIT’s impact on key physiological parameters in obese adolescents. One of the major strengths of this study is its comprehensive approach to synthesizing data from multiple studies, allowing for a more robust evaluation of HIIT’s impact. The inclusion of multiple physiological parameters, rather than focusing solely on weight-related measures, highlights the broader health benefits of HIIT. Notably, the findings demonstrate significant improvements in BFP, VO_2_peak, HDL, and SBP, suggesting that HIIT could effectively improve cardiovascular fitness and metabolic health in this group.

However, several limitations should be acknowledged. First, the selection and screening of studies involved subjective judgment, which may introduce potential bias. Although rigorous inclusion criteria were applied, the relatively small number of included studies limits the generalizability of the findings and increases the risk of publication bias. Additionally, this meta-analysis only included studies published in English, potentially excluding relevant evidence from non-English sources. Another notable limitation is the lack of long-term follow-up in most studies, which hinders the assessment of the sustainability of HIIT-induced physiological changes over time. Furthermore, the scope of health-related outcomes assessed in this review was relatively narrow. While the analysis focused on key indicators such as BFP, VO_2_peak, HDL, and SBP, other clinically relevant markers, such as insulin sensitivity, glucose metabolism, inflammatory cytokines (e.g., CRP), LDL cholesterol, and VO_2_max, were either inconsistently reported or entirely absent. This limited profiling constrains the interpretation of HIIT’s broader metabolic and systemic effects. Future research should incorporate more diverse physiological outcomes and longer follow-up periods to provide a more comprehensive evaluation of the clinical impact of HIIT in obese adolescents.

## 5 Conclusion

This systematic review and meta-analysis provide evidence that HIIT can positively influence health-related outcomes in obese adolescents. Specifically, HIIT was found to significantly reduce BFP, enhance VO_2_peak, increase HDL levels, and lower SBP, although its effect on BMI was negligible. However, notable heterogeneity was observed across studies, particularly in analyses of BMI, VO_2_peak, and BFP, which was partly explained through subgroup analyses based on protocol characteristics such as frequency and session duration. These findings highlight the importance of standardizing HIIT protocols in future interventions.[Fn fn1]

Given the variation in study settings, participant demographics, and geographic contexts, caution should be exercised when generalizing the results to broader adolescent populations. Additionally, important health-related biomarkers such as LDL, insulin sensitivity, and inflammatory markers were either inconsistently reported or absent in the included studies, limiting the scope of conclusions regarding HIIT’s full metabolic impact.

From a practical standpoint, structured HIIT programs that involve moderate session durations (30–60 min) performed at least twice per week appear to yield the most consistent improvements. While HIIT shows promise as a standalone intervention, it may also serve as a complementary approach when combined with traditional physical activity or lifestyle interventions. To support implementation in real-world settings, schools and community health programs could incorporate HIIT into physical education curricula or extracurricular programs using minimal equipment. Clinicians and public health practitioners should consider HIIT as a time-efficient, adaptable strategy when developing exercise prescriptions for obese adolescents. Future research should prioritize protocol standardization and long-term follow-up while also exploring additional physiological and psychosocial outcomes.

## Data Availability

The original contributions presented in the study are included in the article/Supplementary Material, further inquiries can be directed to the corresponding authors.

## Footnotes

1 All publicly available datasets used in this study can be found at: PubMed (https://pubmed.ncbi.nlm.nih.gov/), Web of Science (https://webofscience.clarivate.cn/), Embase (https://www.embase.com/landing?status=grey), and Cochrane Library (https://www.cochranelibrary.com/).
