# Supplementary figures and images for: The effect of high-intensity interval training on health-related outcomes in obese adolescents: a systematic review and meta-analysis
Source: Front Physiol. 2025 Aug 20;16:1609818. doi: 10.3389/fphys.2025.1609818 (PMC12403219; doi:10.3389/fphys.2025.1609818)

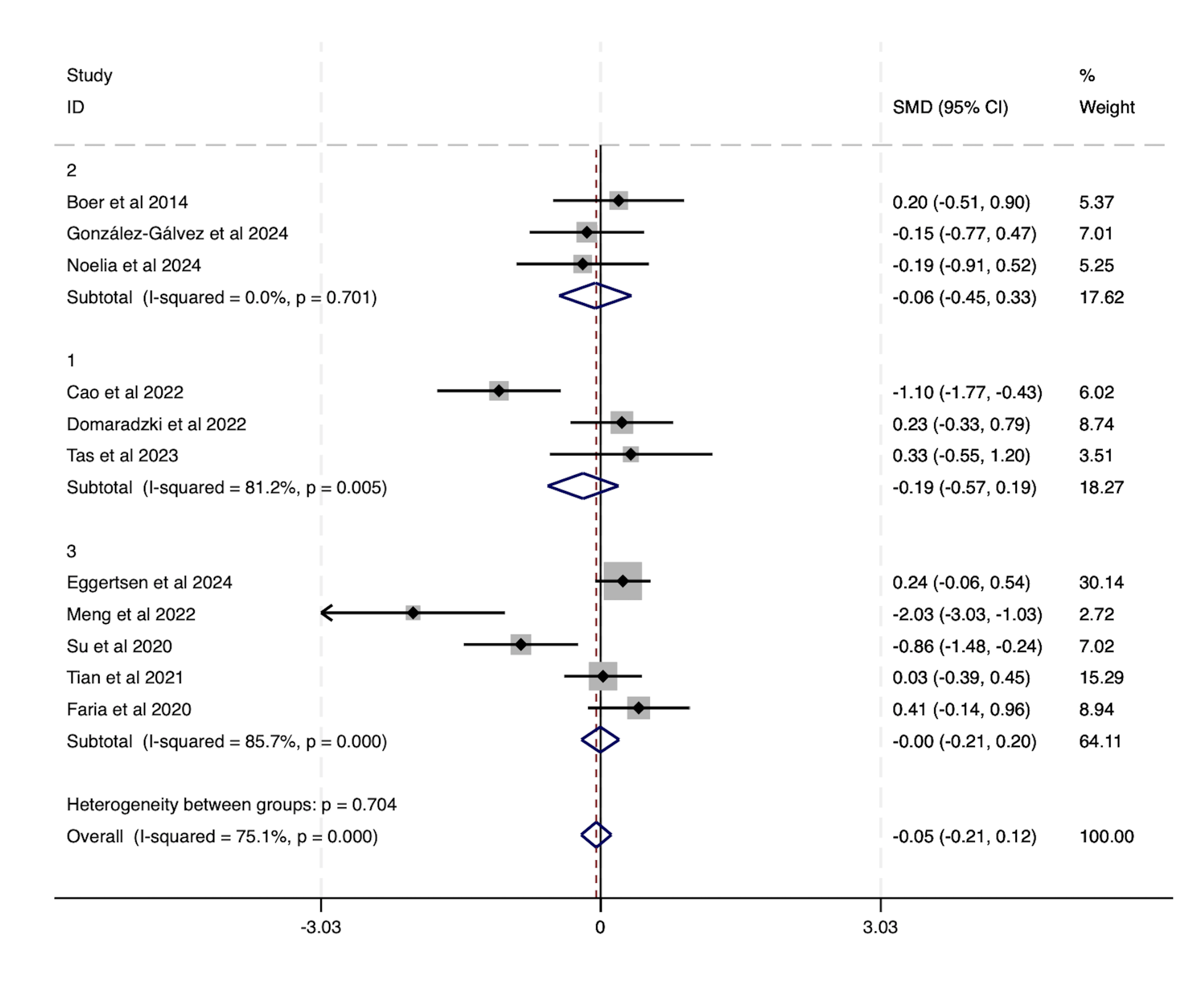

Supplement: Supplementary file 1 [file Image1.PNG]

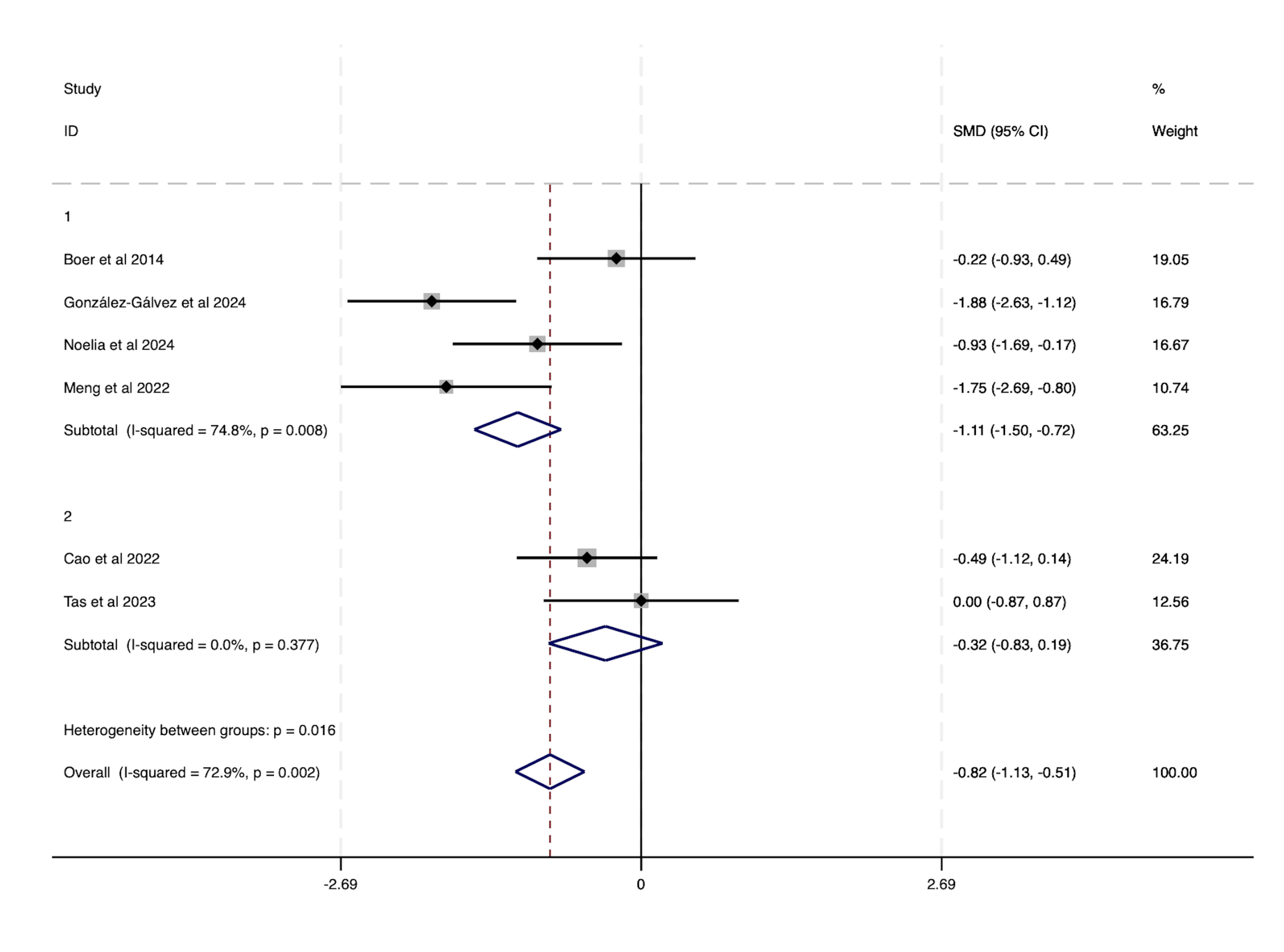

Supplement: Supplementary file 2 [file Image2.PNG]

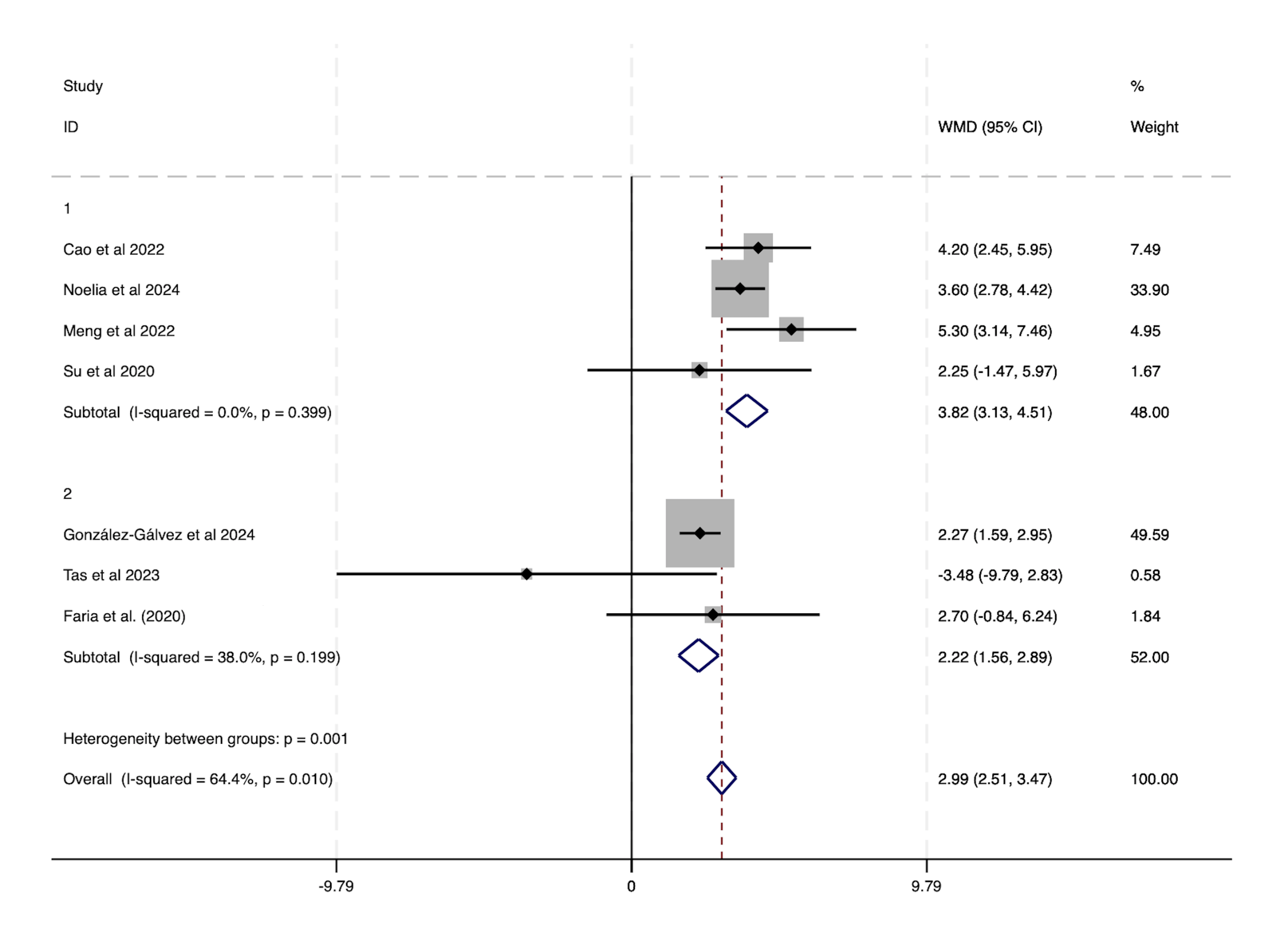

Supplement: Supplementary file 3 [file Image3.PNG]

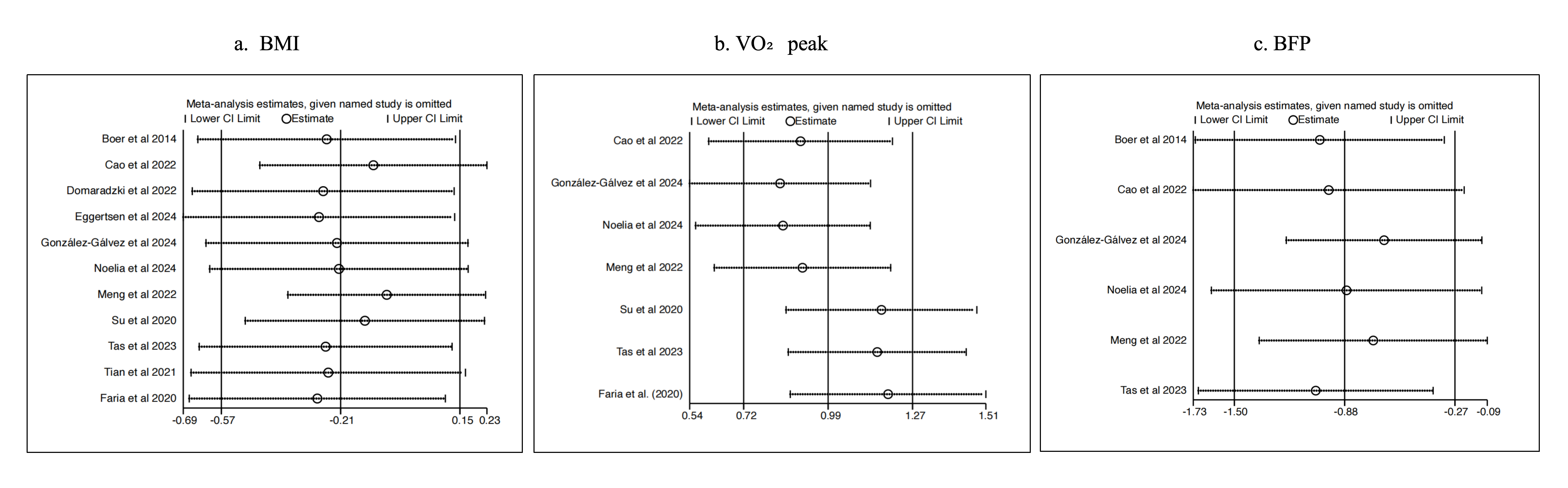

Supplement: Supplementary file 4 [file Image4.PNG]
