# Supplementary material for: The effect of high-intensity interval training on health-related outcomes in obese adolescents: a systematic review and meta-analysis
Source: Front Physiol. 2025 Aug 20;16:1609818. doi: 10.3389/fphys.2025.1609818 (PMC12403219; doi:10.3389/fphys.2025.1609818)
Supplement: Supplementary file 5 [file Table1.docx]

**The effect of High-Intensity Interval Training on Health-related Outcomes in Obese Adolescents: A Systematic Review and Meta-Analysis**

**Gang Xu**

**Supplementary Table S1** Search strategy for **Web of Science Core Collection, PubMed, Cochrane Library, and Embase** databases.

Search strategy for **Web of Science Core Collection** database (inception-December 20, 2024).

| **Category** | **Search terms** |
| --- | --- |
| Population | **#1** (TS=(Obesity) OR AB=(Obesity OR obese OR fat OR corpulence OR adiposis OR overweight)) **#2** (TS=(Adolescent) OR AB=(Adolescent OR adolescents OR adolescence OR youth OR youths OR teenager)) **#3** #1 AND #2 |
| AND Intervention | **#4** (TS=(High-Intensity Interval Training) OR AB=(High Intensity Interval Training OR High-Intensity Interval Trainings OR Interval Training, High-Intensity OR Interval Trainings, High-Intensity OR Training, High-Intensity Interval OR Trainings, High-Intensity Interval OR High-Intensity Intermittent Exercise OR Exercise, High-Intensity Intermittent OR Exercises, High-Intensity Intermittent OR High-Intensity Intermittent Exercises OR Sprint Interval Training OR Sprint Interval Trainings OR HIIT)) |
| AND Study Design | **#5** (TS=(Randomized controlled trials) OR AB=(Randomized controlled trials OR RCTs)) |
| Result | **#6 #3 AND #4 AND #5** 1,524 |

Search strategy for **PubMed** database (inception-December 20, 2024).

| **Category** | **Search terms** |
| --- | --- |
| Population | **#1** "Obesity"[Mesh] **#2** (((((("Obesity"[Mesh]) OR (Obesity[Title/Abstract])) OR (obese[Title/Abstract])) OR (fat[Title/Abstract])) OR (corpulence[Title/Abstract])) OR (adiposis[Title/Abstract])) OR (overweight[Title/Abstract]) **#3** "Adolescent"[Mesh] **#4** (((((("Adolescent"[Mesh]) OR (Adolescent[Title/Abstract])) OR (adolescents[Title/Abstract])) OR (adolescence[Title/Abstract])) OR (youth[Title/Abstract])) OR (youths[Title/Abstract])) OR (teenager[Title/Abstract]) **#5 #2 AND #4** |
| AND Intervention | **#6** "High-Intensity Interval Training"[Mesh] **#7** ((((((((((((("High-Intensity Interval Training"[Mesh]) OR (High Intensity Interval Training[Title/Abstract])) OR (High-Intensity Interval Trainings[Title/Abstract])) OR (Interval Training, High-Intensity[Title/Abstract])) OR (Interval Trainings, High-Intensity[Title/Abstract])) OR (Training, High-Intensity Interval[Title/Abstract])) OR (Trainings, High-Intensity Interval[Title/Abstract])) OR (High-Intensity Intermittent Exercise[Title/Abstract])) OR (Exercise, High-Intensity Intermittent[Title/Abstract])) OR (Exercises, High-Intensity Intermittent[Title/Abstract])) OR (High-Intensity Intermittent Exercises[Title/Abstract])) OR (Sprint Interval Training[Title/Abstract])) OR (Sprint Interval Trainings[Title/Abstract])) OR (HIIT[Title/Abstract]) |
| AND Study Design | **#8** "Randomized controlled trials"[Mesh] **#9** (("Randomized controlled trials"[Mesh]) OR (Randomized controlled trials[Title/Abstract])) OR (RCTs[Title/Abstract]) |
| Result | **#10 #5 AND #7 AND #9** 1,372 |

Search strategy for **Embase** database (inception-December 20, 2024).

| **Category** | **Search terms** |
| --- | --- |
| Population | **#1** obesity:ti OR obese:ti OR fat:ti OR corpulence:ti OR adiposis:ti OR overweight:ti **#2** Adolescent:ti OR adolescents:ti OR adolescence:ti OR youth:ti OR youths:ti OR teenager:ti **#3 #1 AND #2** |
| AND Intervention | **#4** 'high intensity interval training':ti OR 'high-intensity interval trainings':ti OR 'interval training, high-intensity':ti OR 'interval trainings, high-intensity':ti OR 'training, high-intensity interval':ti OR 'trainings, high-intensity interval':ti OR 'high-intensity intermittent exercise':ti OR 'exercise, high-intensity intermittent':ti OR 'exercises, high-intensity intermittent':ti OR 'high-intensity intermittent exercises':ti OR 'sprint interval training':ti OR 'sprint interval trainings':ti OR hiit:ti |
| AND Study Design | **#5** Randomized controlled trials:ti OR RCTs:ti |
| Result | **#6 #3 AND #4 AND #5** 402 |

Search strategy for **Cochrane library** database (inception-December 20, 2024).

| **Category** | **Search terms** |
| --- | --- |
| Population | **#1** MeSH descriptor: [Obesity] explode all trees **#2** (Obesity OR obese OR fat OR corpulence OR adiposis OR overweight):ti,ab,kw **#3 #1 OR #2 #4** MeSH descriptor: [Adolescent] explode all trees **#5** (Adolescent OR adolescents OR adolescence OR youth OR youths OR teenager):ti,ab,kw  **#6 #4 OR #5** |
| AND Intervention | **#7** MeSH descriptor: [High-Intensity Interval Training] explode all trees **#8** (High Intensity Interval Training OR High-Intensity Interval Trainings OR Interval Training, High-Intensity OR Interval Trainings, High-Intensity OR Training, High-Intensity Interval OR Trainings, High-Intensity Interval OR High-Intensity Intermittent Exercise OR Exercise, High-Intensity Intermittent OR Exercises, High-Intensity Intermittent OR High-Intensity Intermittent Exercises OR Sprint Interval Training OR Sprint Interval Trainings OR HIIT) **#9 #7 OR #8** |
| AND Study Design | **#10** MeSH descriptor: [Randomized controlled trials] explode all trees **#11** (Randomized controlled trials OR RCTs):ti,ab,kw **#12 #10 OR #11** |
| Result | **#13 #3 AND #6 AND #9 AND #12** 1,479 |
